# Quantitative proteome profiling stratifies fibroepithelial lesions of the breast

**DOI:** 10.18632/oncotarget.27889

**Published:** 2021-03-02

**Authors:** Aviral Kumar, David S. Nayakanti, Kiran K. Mangalaparthi, Veena Gopinath, Nandyala Venkat Narsimha Reddy, Krishna Govindan, Geeta Voolapalli, Prashant Kumar, Lekha Dinesh Kumar

**Affiliations:** ^1^Cancer Biology, CSIR-Centre for Cellular and Molecular Biology, Hyderabad, Telangana, 500007, India; ^2^Institute of Bioinformatics, Discoverer Building International Tech Park, Whitefield, Bangalore, 560066, India; ^3^Department of Surgery and Pathology, Gandhi Hospital, Secunderabad, 500025, India; ^4^Department of Pathology, Government Medical College, Thiruvananthapuram, Kerala, 695011, India; ^5^Manipal Academy of Higher Education, Manipal, Karnataka, 576104, India; ^*^Authors share equal first authorship; ^#^Authors share equal second authorship

**Keywords:** breast tumors, fibroepithelial lesions, phyllodes, iTRAQ, quantitative proteomics

## Abstract

Breast fibroepithelial lesions (FELs) include heterogeneous pathological tumors, involving indolent fibroadenoma (FAD) to potentially aggressive phyllodes tumors (PTs). The current grading system remains unreliable in differentiating these tumors due to histological heterogeneity and lack of appropriate markers to monitor the sudden and unpredictable malignant transformation of PTs. Thus, there exists an imminent need for a marker-based diagnostic approach to augment the conventional histological platform that could lead to accurate diagnosis and distinction of FELs. The high- throughput quantitative proteomic analysis suggested that FAD and PTs form distinct clusters away from borderline and malignant though there exist marked differences between them. Interestingly, over-expression of extracellular matrices (ECM) related proteins and epithelial-mesenchymal transition (EMT) markers in borderline PTs led us to hypothesize a model of deposition and degradation leading to ECM remodeling and EMT acquisition triggering its malignant transformation. We also identified three candidate biomarkers such as MUCL1, HTRA1, and VEGDF uniquely expressed in FAD, borderline, and malignant PTs, respectively, which were further validated using immunohistochemistry. The present work shed light on a brief mechanistic framework of PTs aggressive nature and present potential biomarkers to differentiate overlapping FELs that would be of practical utility in augmenting existing diagnosis and disease management for this rare tumor.

## INTRODUCTION

Fibroepithelial lesions (FELs) of the breast are a group of biphasic tumors that are highly heterogeneous in terms of their morphological as well as biological features. FELs include well- defined fibroadenomas (FADs) and phyllodes tumors (PTs), with intersecting histologic attributes but varying clinical implications and outcomes [1, 2]. FADs are widespread tumors accounting for 68% of all breast masses and 44–94% of biopsied breast lesions [3]. However, PTs are rare tumors and account for only < 1% of all breast neoplasms [4], and being highly aggressive requires wide local excision with negative margins. World Health Organization has classified PTs into benign (58%), borderline (12%) and malignant (30%) tumors based on the histopathologic features of stromal components and leaf-like patterns around epithelial lined spaces [5]. PTs are rapidly growing tumors that can often be misdiagnosed, leading to multiple diagnostic complications and invasive procedures [6]. Once these tumors become metastatic, the overall survival decreases to 30 months [7] and correlates with the increased risk of recurrence and rapid deterioration [8]. Conventional therapeutic modalities have not been adequate to improve progression-free survival in high-grade PTs that might warrant a radical mastectomy [9]. Conventionally even though PTs have been considered a disease of middle-aged (around 40 years), cases have been reported recently in young adolescent females in whom fibroadenoma was found to be more prevalent. Thus, molecular characterization of PTs is essential for careful evaluation and distinguishing between the different grades for better clinical outcome and management of this deadly disease.

Owing to the histological heterogeneity within PTs, the accurate diagnosis is one of the major challenges which is paramount for disease management and choice of treatment. A commonly encountered complication in diagnosis is the differentiation of FADs and benign PTs primarily contributed by the overlapping histologic and morphological characteristics between these lesions. It has been previously reported that the sensitivity of Fine needle aspiration cytology (FNAC) and needle core biopsy (NCB) in differentiating PTs and FAD is approximately 40% and 63% only [10]. Diagnostic uncertainty exists between benign or borderline tumors and FAD due to moderate stromal cellularity and atypia, epithelial hyperplasia, increased mitosis, and circumscribed borders [11]. Tremendous efforts have been made to characterize the fibroepithelial lesions extensively at the genomic level. A study by Tan *et. al*. (2016) identified several recurrently mutated genes unique to FAD and PTs, and several protein markers have also been investigated previously for their diagnostic utility and association with histological grade in FELs [12]. However, not much effort has been made to identify potential diagnostic biomarkers that could improve the diagnostic practice to classify PTs and differentiate them from FADs. The comprehensive proteomic alterations differentiating these FELs have not been reported so far.

Recent advances in mass spectrometry-based proteomics platforms have revolutionized the feasibility of unbiased protein identification and quantification at greater depths [13]. To this end, we employed iTRAQ based quantitative proteomics of FELs to extensively characterize the proteomic alterations across these tumors in order to identify potential biomarkers and distinctly stratify these overlapping tumors. The comprehensive proteomics approach will further enhance the understanding of this tumor biology and dynamics at the molecular level and can further aid in identifying reliable protein-based markers for improving clinical management of the disease.

## RESULTS

### Quantitative proteomic profiling of fibro-epithelial lesions of the breast

**Figure 1:**
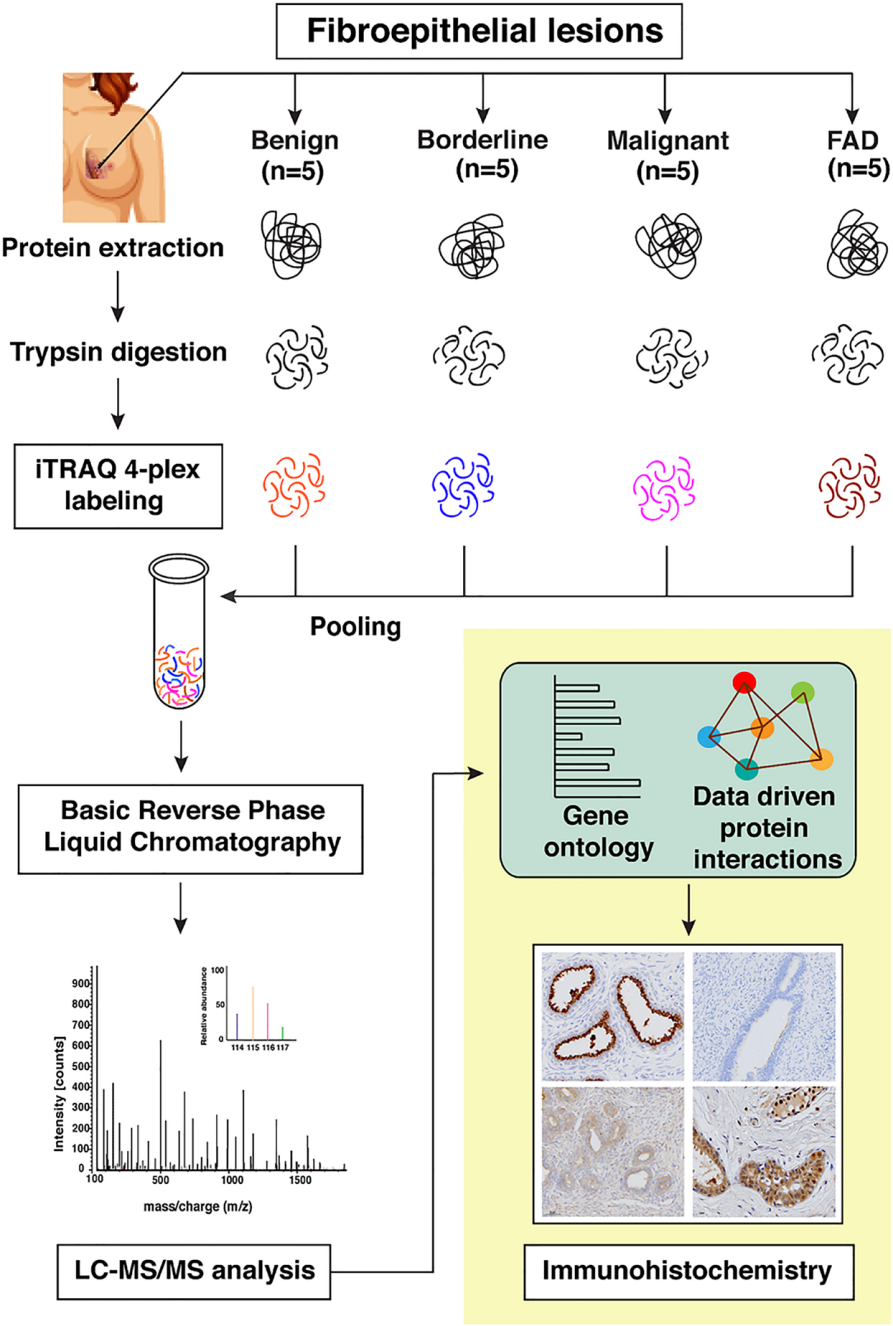
Schematic workflow for the identification of differentially expressed proteins across FELs using iTRAQ based quantitative proteomics approach. The expression levels of candidate proteins were validated using immunohistochemistry.

Quantitative global proteomics was conducted on FAD and PTs (benign, borderline, and malignant) FFPE sections, as illustrated in Figure 1. To increase the reliability of analyses, we included technical replicates in our study. The MS data were processed and searched against databases by SEQUEST and MASCOT algorithms using the Proteome Discoverer 2.1 platform. A strict FDR of 0.01 (1% cut off) was defined for identifying the confident peptide spectral matches where 7717 proteins were identified in the initial discovery phase with 6837 proteins quantified in at least two replicates shown in Supplementary Tables 2 and 3.

### Distinct proteomic profiles distinguish FAD and benign PTs

**Figure 2:**
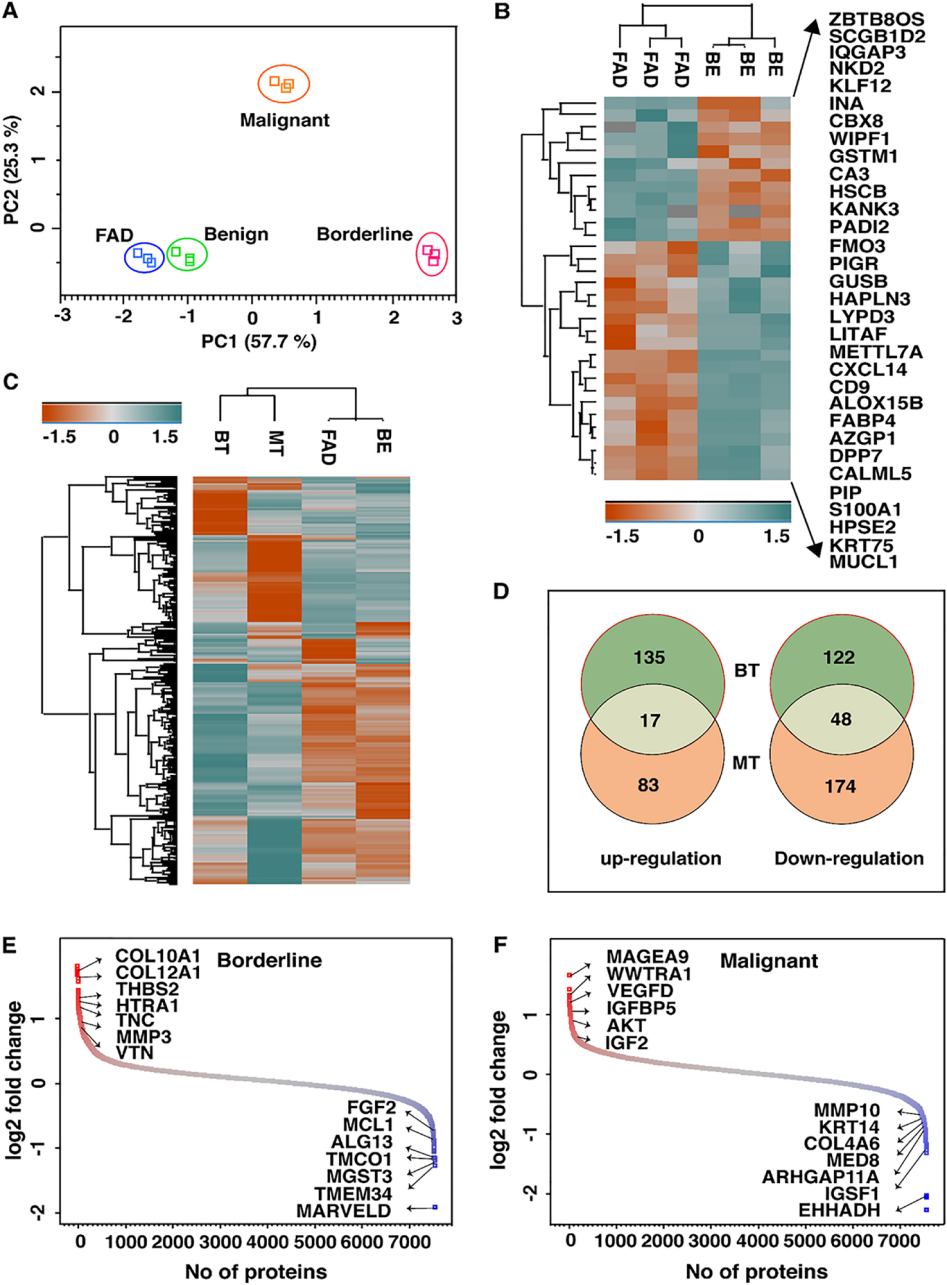
(**A**) Principle component analysis of quantified proteins reveals distinct separation of FELs subtypes where BE and FAD clustered together being mostly as indolent tumors. (**B**) List of proteins that are significantly dysregulated in FAD as compared to BE PTs. (**C**) Heatmap of dysregulated proteins (1.5 folds) in FAD, BT and MT as compared to BE PTs. (**D**) Venn diagram showing unique and common dysregulated proteins in BT and MT PTs. Distribution of log 2- fold changes of total proteins in (**E**) BT vs BE and (**F**) MT vs BE; red and blue dots represents up and down regulated proteins by 1.5 folds respectively.

To identify the proteomic profiles that overlap among FELs, we conducted a principal component analysis of the protein expression matrix. Segregation of all the four cohorts (FAD, benign, borderline, and malignant) was observed with technical replicates grouping together, demonstrating the high reproducibility of MS quantification (Figure 2A). Interestingly, we observed FADs and benign PTs clustered together compared to borderline and malignant ones, albeit with overlapping protein expression profiles. Further investigation led to the identification of 32 proteins that were differentially regulated in FAD as compared to benign PT (*p*-value =< 0.05) (Figure 2B; Supplementary Table 4). MUCL1 was the most overexpressed protein observed in FAD as compared to benign PT (3.2-fold). PIGR, CXCL13, CXCL14, and LYPD3 were among other highly overexpressed proteins and TNC, SCGGB1D2, KANK3, KLF12, HSCB, GK, and, EHHADH were the significantly down-regulated proteins in FAD (>2 fold) compared to benign PT.

### Proteomic profiles differentiate borderline and malignant PTs from benign

Interestingly, borderline and malignant PTs clustered distantly apart from benign in principal component analysis, suggesting significant malignant features of Phyllode tumor. Therefore, further comparison of the proteomic profiles of high-grade PTs (borderline and malignant) with low-grade PTs (benign) were made to investigate differentially expressed proteins in PTs (Figure 2C). A total of 323 (153 up-regulated and 170 down-regulated) and 324 (100 up-regulated and 224 down-regulated) specific proteins were identified in borderline and malignant tumors respectively, compared to benign tumour. The distribution of these proteins between borderline and malignant PTs is shown in Figure 2D. Proteins with at least 1.5-fold or higher were considered as differentially and significantly expressed proteins. With these criteria, a set of 162 and 150 proteins were found in borderline PT and malignant PT, respectively (Supplementary Tables 5 and 6).

Among these, the significant ones included ACAN, INHBA, COL11A1, THBS2, APOC1, APOB, TAZ, HTRA1, FGG, and MAGEA4 among the up-regulated proteins and MARVELD, TMEM134, TMCO1, TRIM26, GFOD2, UQCR10, ADH1C, MCL1, CHRAC1, and SPRYD3 among the down-regulated proteins reported in borderline PT (> 2 fold) (Figure 2E). In malignant PT, MAGEA9, MOXD1, WWTR1, APOC1, UCHL1, VEGFD, PPIC, KNG1 were significantly over-expressed and SCGB1D2, HSCB, GK, KLF12, TRIM26, ZBTB80S, CA3, ARHGAP11A, KDM4B, and AGR2 were down-regulated with more than two-fold change (Figure 2F). Notably, the overlap of the expressed proteins was limited to 67 proteins that were common to both borderline and malignant tumors; important ones included ATG12, SFRP4, CDKN1C, PON1, SOD3, LAD1, MST1R, FSTL1, PIR, MAGEA4, TPM1, ADAMTSL1, FGG, and LBH. Several proteins reported to be dysregulated in other cancers including breast cancer were also observed in borderline and malignant PTs. Some of the most significant ones included HTRA1, COL10A1, COL12A1, THBS2, ITGA2B, FGF2, MARVELD and TMCO1 in borderline tumors and VEGFD, WWTR1, IGFBP5, AKT, CRABP1, KLF1, COL4A6, IGSF1, MMP10 and MED8, in malignant tumors.

### Distinct biological processes involved in borderline to malignant transformation

To further investigate the biological processes associated with over-expressed (1.5-fold up) proteins in borderline and malignant tumors, we employed enrichment analysis in gene ontology terms (Supplementary Figure 1). While extracellular reorganization, cell adhesion, proteolysis, immune complement activation, platelet degranulation, and inflammation were the major biological processes enriched in borderline tumors, oxidative phosphorylation linked to metabolic reprogramming in cancer, SRP-dependent co-translational protein targeting to membrane, negative regulation of apoptosis, positive regulation of protein kinase B signaling, intracellular signal transduction, translation initiation, and mRNA splicing via spliceosome were found to be linked with malignant tumors.

### ECM remodeling as the major enriched biological process in borderline tumors

**Figure 3:**
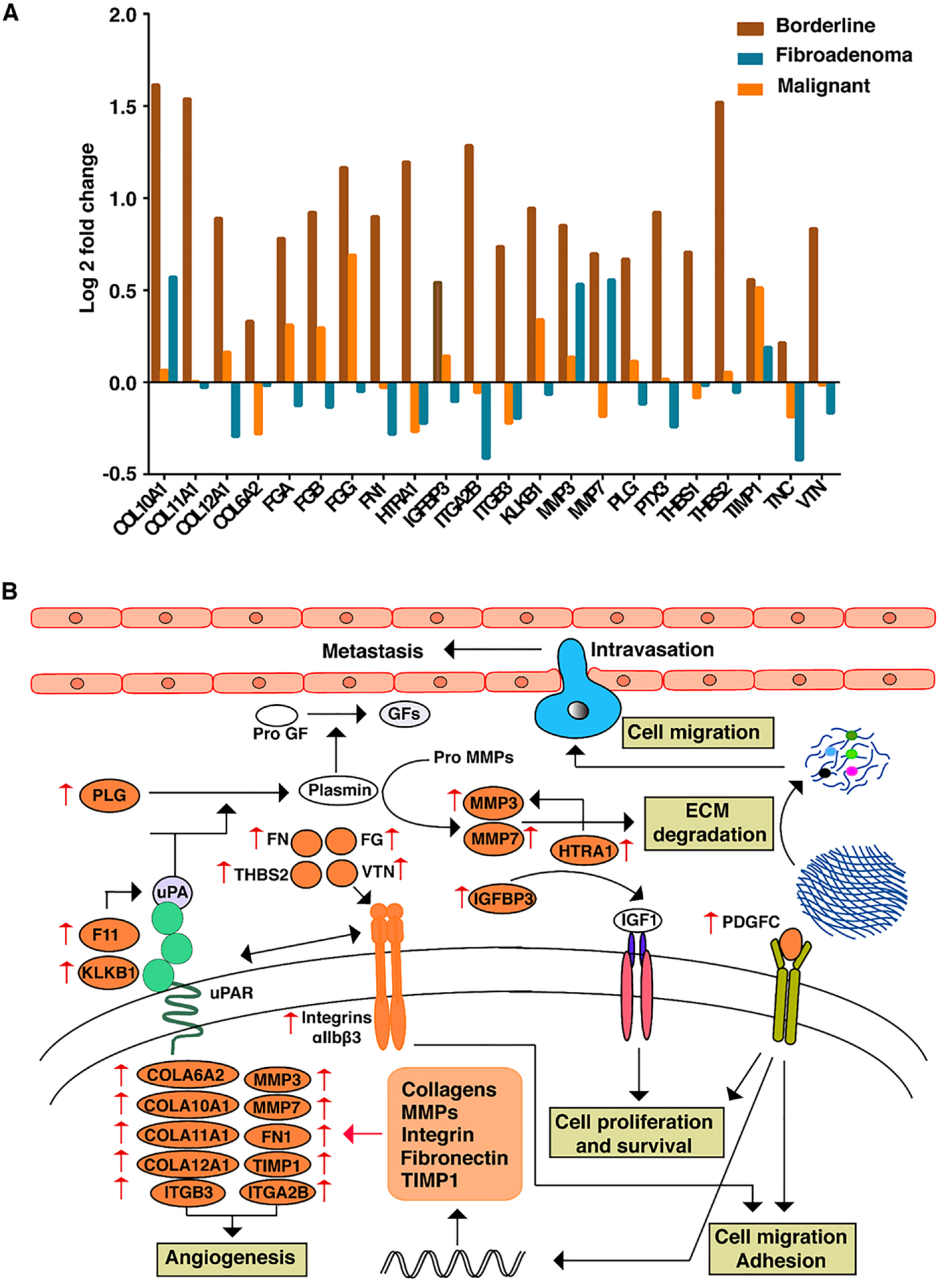
(**A**) Expression patterns of proteins involved in ECM remodelling across FEL subtypes. (**B**) Data driven comprehensive network map of ECM proteins and their interactions which are enriched in BT subtype.

To investigate further the biological processes that might be regulating the process of malignant transformation in borderline PTs, we carried out DAVID gene ontology term analysis using the set of 153 overexpressed proteins in borderline PT as compared to benign PT. Proteolysis, platelet degranulation, negative regulation of endopeptidase activity, blood coagulation, and extracellular matrix organization were the major biological processes enriched in borderline tumors (Figure 3A). Among the biological processes enriched, several proteins that played an important role in extracellular matrix remodeling were uniquely dysregulated in borderline tumors. ECM reorganization plays a crucial role in the acquisition of EMT phenotype as shown in several cancers. Twenty-two proteins known to be involved in the extracellular matrix (ECM) organization and disassembly, including several collagens (COL10A1, COL11A1, COL12A1, and COL6A2), ECM proteins (FN1, THBS1, THBS2, VTN, PLG, and HTRA1), fibrinogens (FGA, FGB, and FGG), ECM degrading enzymes (MMP3 and MMP7) and integrins (ITGA2B and ITGB3) were identified. LAD1, ECM2, GFOD1, FBLN5, ADAMTSL4, COL21A1 were down-regulated in borderline tumors. Also, we have observed several proteins, such as PTX3, COL6A2, IGFBP3, TNC, TIMP1, SERPINF1, PDGFC, and BGN, that are implicated in EMT acquisition that were over-expressed in borderline tumors, suggesting that ECM remodeling and acquisition might play a significant role in the progression of borderline PTs. HTRA1 was 2.2-fold overexpressed in the borderline PT and is a known regulator of ECM degrading enzymes and initiate early events in intravasation of the tumor cells into circulation. A schematic representation of the extracellular matrix degradation and EMT proteins interaction pathway is shown in Figure 3B.

### Malignant tumors are abundant in proteins involved in intracellular oncogenic signaling cascades

To identify the unique proteins that are deregulated exclusively in malignant PTs, we segregated the 91 proteins that do not belong to other categories. We found that proteins such as EDNRA, MB, POSTN, VEGFD, IGF2, and KNG1 that are potent factors in hypoxia and platelet degranulation, were in abundance in malignant PTs. Also, it was found to be enriched with proteins involved in various cancer-associated intracellular signaling cascades including angiogenesis (VEGF, FGF2, VCAN, and THBD), oxidative phosphorylation (ATPB1, RHOT1, MGST3, UQCRQ, UQCR10, POLR2F, NDUFA7, and NDUFS8), enhanced activation of translation machinery (RPL8, RPL18A, RPL21, RPL24, RPL27A, RPL29, RPL31, RPL32, RPL34, RPL35, RPS23, MRPL4, and MRPL55), transcription factors (WWTR1, MED1 and MED 25), and E2F targets (H2AFX, CKS1B, CDKN2C, and SNRPB) etc. Growth factors like VEGFD, FGF2, IGF2, EGFR, and many oncogenic proteins like IGFBP5, AKT, PLCL2, PIK3CD, FER, CRABP1, and CRABP2 that modulated cell proliferation, survival, migration, and angiogenesis showed elevated expression in malignant tumors. Interestingly, many vital anti-apoptotic proteins like MCL1, HAX1, BNIP2, PSMD10 and SQSTM1 were also up-regulated in malignant PTs indicating the metastatic state of the tumor. We observed the over-expression of proteins like MED1, SQSTM1, RHOT1, TPD52L2, FUT8 and TLK1 that impacted cellular homeostasis contributing to tumor growth and metastasis. High expression of ALDH1 (ALDH1A2 and ALDH1 A3), a mesenchymal marker in malignant tumors, indicated mesenchymal stem cell-like features that would expedite the transformation of these tumors into a liposarcoma, rhabdomyosarcoma, and osteosarcoma as reported earlier [14]. It was noticed that AKT3 was overexpressed while INPP4B, a negative regulator of AKT, was downregulated in malignant tumors suggesting that AKT might play a crucial role in malignant transformation and aggressive behavior of phyllodes.

### Validation of biomarkers for FAD, borderline and malignant PTs

**Figure 4:**
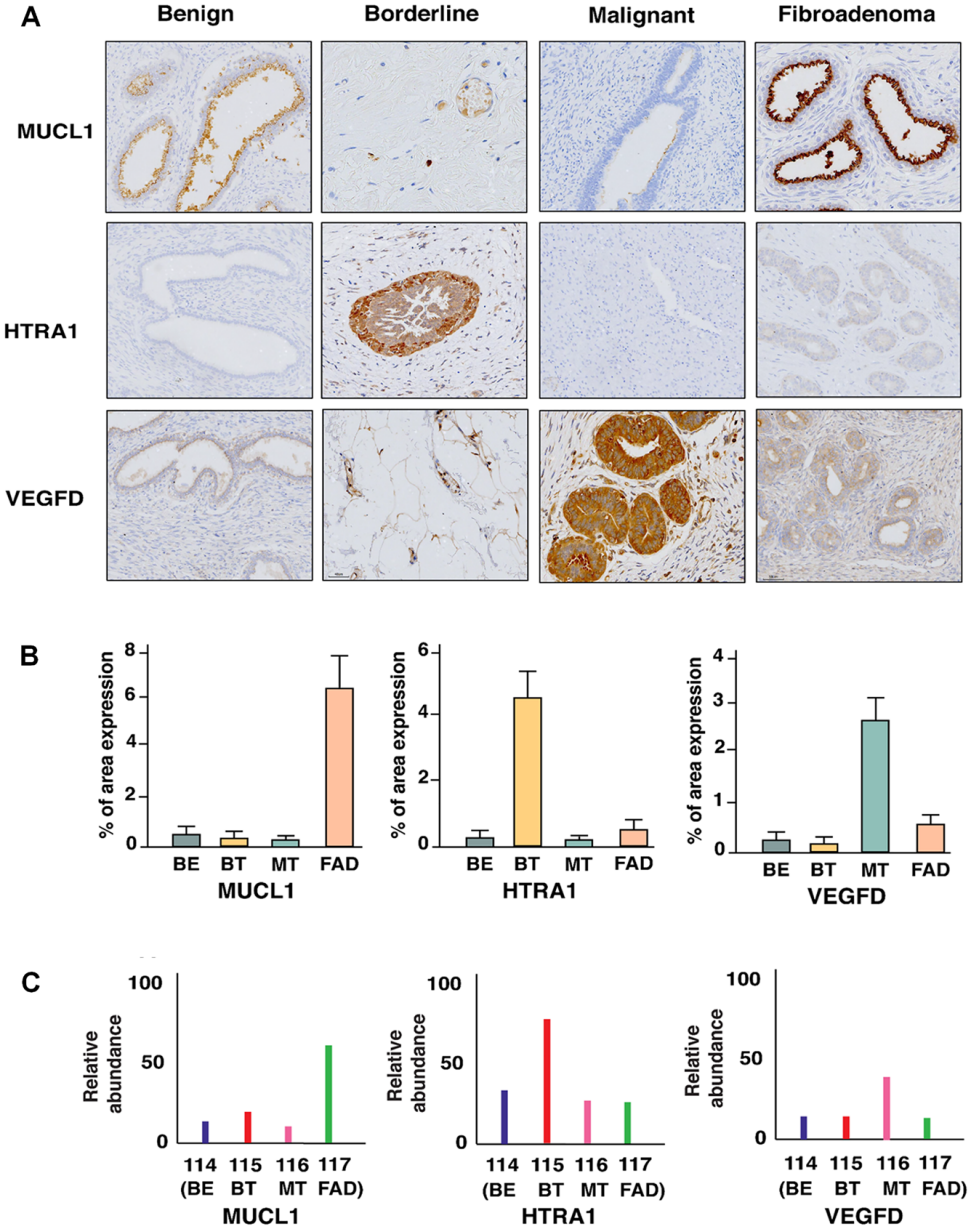
(**A**) Immunohistochemical staining of MUCL1, HTRA1, and VEGFD which are uniquely overexpressed in FAD, BT, and MT respectively. (**B**) Percentage of area expression and (**C**) relative abundance of MUCL1, HTRA1, and VEGFD in FAD, BT, and MT respectively.

Our global in-depth proteomic analysis enabled us to identify candidate proteins uniquely dysregulated in each of the FELs. These proteins could serve as novel potential markers to classify these tumors unambiguously which, clinically show overlapping features. To that end, we carried out immunohistochemical validations for various proteins involved in FELs pathogenies (Supplementary Figure 2). Three proteins MUCL1, HTRA1, and VEGFD which showed unique and significant overexpression in fibroadenoma, borderline, and malignant phyllodes, respectively, were selected based on the confidence of MS data and independent validation of these proteins was carried out in clinical samples using immune-histochemistry. The validation and quantitative analysis of the results on five biological replicates of each FEL group were in concordance with our MS-based proteomic analysis (Figure 4 and Supplementary Figures 3–5). MUCL1 was seen to be overexpressed in the ductal epithelial lining of fibroadenoma patients, while; a scanty expression was seen in the secretory epithelial linings of benign phyllodes. However, borderline and malignant tumors were devoid of any expression. High expression of VEGFD was observed in the hypercellular stromal fragments, epithelial and myoepithelial lined spaces of malignant sections compared to the fibroadenomas, while the borderline sections exhibited increased levels of HTRA1 proteins, especially in the stromal regions in contrast to the other category of phyllodes.

## DISCUSSION

Despite our understanding of breast tumor pathophysiology, FELs remains challenging due to its unpredictable clinical behavior and high risk of rapid metastasis. Though there are several comprehensive genetic studies of PTs, an unmet clinical need exists for accurate diagnosis, prognosis, and stratification of FELs, which could fundamentally improve the disease management strategies. Even though benign PTs and fibroadenoma pose significant diagnostic challenges, borderline and malignant prognostic factors are of prime importance due to their aggressive and rapid progressive nature.

In recent times, MS-based quantitative proteomic analysis has emerged as a powerful platform to carry out in-depth proteome profiling across different cancers [15]. We conducted a global proteomics-based comparative study to distinguish the comprehensive proteomic profiles of FELs and identify the unique proteomic expressions in each group. Based on our proteomic data, we not only classified sets of significant and unique proteins for each group but also validated the expressions using immunohistochemistry. To the best of our knowledge, this is the first extensive global proteomic profiling carried out in FELs. Further validation of these proteins could lead to the development of a robust set of potential markers that could be used to assess the clinical characteristics of these FELs without ambiguity.

This study revealed that FAD and benign PT, being entirely two different entities of FELs, manifest similar proteome profiles that demarcate them from other FELs. Both lesions arise from intralobular fibrous tissue and display coinciding histological features. The distinction between these two lesions is clinically critical due to varied surgical management ranging from observation to wide local excision [16]. Notably, some proteins which were reported earlier for breast cancer were also found to be dysregulated in FADs and benign PTs though with different fold changes. For example, MUCL1 that was found to be over-expressed in FAD, was also observed in the epithelial lining of the duct in breast carcinomas. This observation was further validated by immunohistochemistry. Higher expression of MUCL1 is usually associated with a higher grade of breast cancer and is known to be regulated by HER2 [17]. Though its functional role in breast cancer has been elucidated well, its role in FAD is yet to be explored. Similarly, as observed in our study, over-expression of PIP (Prolactin inducible protein) has also been reported in all breast cancer subtypes [18]. Lower levels of PIP was associated with infiltrating breast carcinoma than *in-situ* carcinomas suggesting that, FAD being benign in nature has high levels of PIP and expression decreases with higher stage and grade of breast cancer. Considering these observations, it seems likely that there exist molecular similarities between FAD and benign PTs.

The distinction between the progressive stages of PT, (borderline and malignant tumor class) is of prime importance due to their rapid progression to the aggressive metastatic stage. Since borderline PTs exist as an intermediate state between benign and malignant tumors with invasive potential, we sought to identify novel markers for recognizing these tumors. Borderline PTs were enriched with proteins that are involved in ECM remodeling. Though we cannot rule out the possibility that other pathways are also involved in the transformation and rapid progression of phyllode tumors, ECM helps in intricate cross-talk so as to modulate signals governing cellular phenotypes. This includes cell proliferation, survival, motility, and adhesion properties, which are known to be highly deregulated during cancer progression [19]. This study also indicated that several collagen proteins like COL10A1, COL11A1, COL12A1, and COL6A2, previously reported to be expressed in different cancer types during metastasis, have higher expression in borderline PT compared to other FELs. Previous studies have reported that the overexpression of COL10A1 is implicated in promotion of cell proliferation, migration, invasion, and is usually associated with poor prognosis in colorectal cancer (CRC) [20] and non-small cell lung carcinoma [21], respectively. Similarly, high expression of COL12A1 was found in breast cancer, gastric cancer, and CRC [22], while COL6A2 was over expressed in ovarian cancer and promoted metastasis contributing to poor survival in patients [23]. In addition to collagens, several other proteins associated with ECM such as FN1, THBS1, THBS2, VTN, PLG, HTRA1, TNC, FGA, FGB, and FGG also showed high expression in borderline PTs suggesting increased density and altered composition of ECM thus contributing to borderline-malignant transition. These over expressed ECM proteins may interact with integrins to modulate signals involved in malignant transformation [24].

Higher expression of integrins ITGA2B and ITGB3 was also observed in borderline PT samples. One of the early events associated with cancer cell invasion and metastasis is the proteolytic degradation of ECM that is predominantly mediated by MMPs and urokinase-type plasminogen activator (uPA) system, which in turn activates plasminogen [25]. Plasminogen and upstream activators like coagulation factor XI and kallikrein, as reported earlier, were also upregulated in borderline tumors in this study [26]. ECM degradation is known to induce cancer cells to invade adjacent tissues and intravasate into blood vessels during metastasis. In borderline PT, MMP-3 and MMP- 7, that are known to promote tumor cell invasion, were highly expressed triggering EMT into malignant state [27]. MMP7 promotes tumor invasion by activating nuclear factor kappa B ligand while, MMP2 and MMP9 enhance cell proliferation by cleaving heparin thus favoring cancer cell survival [28]. The over expression of these MMPs could function as powerful machinery to degrade ECM and facilitate tumor invasion, an indication of aggressive malignant behavior. Another protein we identified was THBS-2, the level of which was elevated in borderline PTs. This was positively correlated with the migration of lung cancer cells through integrin/FAK/Akt/NF-κB signal transduction in many studies [29]. Most interestingly, ECM degrading enzyme, HTRA1, was observed to be significantly expressed in borderline PT. It is a secreted serine protease that degrades ECM by cleaving fibronectin and the released fragments in turn regulate cell migration and invasion by enhancing MMP-3 expression. HTRA1 regulates the availability of FGF and IGF by cleaving proteoglycans and IGF binding proteins respectively; IGFBP-3 is also over expressed in borderline PTs, which is cleaved by HTRA1 to release IGF1 [30]. These observations clearly indicated HTRA1 as a promising candidate unique to borderline PTs. Overall, the cross-talks between over expression/activation of MMPs, plasminogen activation, and integrin signaling, functions as a powerful machinery to degrade ECM and facilitate tumor cell invasion and metastasis. Targeting integrin signaling and MMP activation could be a pivotal strategy to regress and arrest the progression of borderline PTs.

Fundamental delineation of the EMT phenotype and their intricate cross talks is essential to understand the mechanistic cues for its malignant transformation. Since over-expression of ECM proteins and ECM remodeling is mostly associated with the acquisition of EMT phenotype in many cancers, we further assessed the levels of EMT proteins in PTs. Interestingly, borderline PTs were enriched with several EMT markers including PTX3, TNC, IGFBP3, and PDGFC that promote tumor growth by acting as mitotic and survival factors. PDGFC promotes tumor growth by acting as mitogenic and survival factors and induces angiogenesis in VEGF independent pathway [31]. PTX3 enhances the expression of vimentin, fibronectin, and MMPs including MMP3 and MMP7 that are reported to drive EMT accelerating cancer progression in hepato-cellular carcinoma [32]. Fundamental delineation of their cross talks is essential to understand the exact mechanism for its malignant transformation. These observations of enhanced expression of such factors in borderline PTs help them to modulate survival and invasive capabilities, by increased angiogenesis through ECM degradation and remodeling in concordance with EMT acquisition. Also, we identified platelet degranulation as an active component in malignant PTs and it is known that components of the hemostatic system contribute to the process of angiogenesis during malignancy [33]. VEGFD, a member of the platelet- derived growth factor/vascular endothelial growth factor (PDGF/VEGF) family, implicated to be active in angiogenesis, lymphangiogenesis, and endothelial cell growth [34], was also found to be over expressed in the malignant PTs. This secreted protein undergoes a complex proteolytic maturation, generating multiple processed forms that bind and activate VEGFR-2 that further regulate angiogenesis [35, 36]. Recent studies have demonstrated that VEGF receptors are expressed in several cancer cells types and may dictate proliferation and invasion of these cells [37, 38]. Taken together, our study has established an important functional role of biological process involved in rare FELs which might provide cues in assessing the clinical outcome of the disease.

In conclusion, this study provided a comprehensive profile of differentially regulated proteins across various subtypes of FELs. The presence of extensive ECM proteins and EMT markers led us to hypothesize a model of deposition and degradation of these proteins thus triggering ECM remodeling and EMT acquisition in borderline PTs leading to its malignant state. Enrichment of platelet degranulation factors in malignant PT indicates active angiogenesis during this transformation. Herein, our initial findings suggest that MUCL1, HTRA1, and VEGFD can be used as potential proteomic markers that could augment existing diagnosis, and help in monitoring the progression of the disease. Further characterization of FELs using different omics platforms would help in better understanding of the cellular and molecular events that would help in understanding the disease dynamics and thus better management of the disease.

## MATERIALS AND METHODS

### Clinical sample collection

The samples were obtained from the Department of Surgery & Pathology, Gandhi Medical College, Secunderabad, and Government Medical College, Trivandrum, India. This study was approved by the Institutional review board (IEC 47/2016), and ethics committee of respective hospitals and Institutions and all procedures were performed following the declaration of Helsinki, after obtaining written informed consent from the patients, parents/guardians (in case of a minor). A cohort of five independent biological replicates in each category (benign, borderline, malignant PTs, and FAD) was considered for the proteomic analysis, and the details of these patients are shown in Supplementary Table 1. Moreover, additional five independent biological replicates were taken for biomarker validation phase studies. Tissue specimens after surgery were collected in RNA later and stored at 40C overnight followed by storage at –800°C. Samples were extracted from FFPE sections after being confirmed by a pathologist before proteomic analysis.

### Protein extraction

FFPE sections from each block were deparaffinized using xylene for 30 minutes, followed by rehydration with 90% and 70% ethanol for 30 minutes each before washing in water for half an hour. The samples were transferred into tissue lysis buffer (4% SDC in 100 mM Tris- HCL) and incubated at 90ºC for about 1 hour, followed by 30 minutes incubation at –80ºC. The samples were sonicated for five cycles at 60% amplitude on ice. The process of heating, freezing, and sonication is repeated twice. Protein estimation was done by BCA assay, and an equal amount of protein was taken from each group for further analysis.

### In-solution trypsin digestion, iTRAQ labeling, and bRPLC fractionation

Individual samples were subjected to reduction using reduction/alkylation buffer (100 mM TCEP and 400 mM 2-chloroacetamide) at 45 ºC for 10 minutes, followed by overnight digestion using trypsin from Promega (1:50 of the enzyme to the substrate in 0.05% AcOH and 2 mM CaCl2) at 37 ºC [39]. Peptides from each sample were labeled using the iTRAQ-4plex label kit as per the manufacturer’s instruction (Catalog # 4352155, AB SCIEX), and the subsequent labelled peptides from all iTRAQ channels were pooled and then subjected to basic Reverse Phase HPLC fractionation (bRPLC) [40] using the 1260 Infinity HPLC system (Agilent, Santa Clara, CA, USA). The pooled peptide mixture was reconstituted in solvent A (10 mM TEABC in water; pH 8.5) and separated on Waters X-bridge reverse phase C18 column (4.6 × 250 mm, 5 μm; Waters, Milford, MA, USA) using a gradient of 3% solvent B (10 mM TEABC in 90% acetonitrile; pH 8.5) to 50% solvent B over 50 minutes. A total of 96 fractions were collected, which were concatenated into 12 fractions and dried using speed vac for LC-MS/MS analysis.

### LC-MS/MS analysis

Each fraction was suspended in 0.1% formic acid and subjected to LC-MS/MS analysis in technical triplicates. LC-MS/MS analysis was carried out using Q-Exactive HF-X mass spectrometer (Thermo Scientific, Bremen, Germany) connected to an online chromatography platform Dionex Ultimate RSLC 3000 system (Thermo Scientific, Bremen, Germany). Peptides were separated using a two-column setup in which the peptides were initially loaded on a trap column (Thermo Scientific, Acclaim PepMap 100, 75 μm × 2 cm, 3 μm C18 100A˚) using solvent A (5% Acetonitrile, 0.1% Formic acid) and separated on 50 cm analytical column (Thermo Scientific, Acclaim PepMap RSLC, 75 μm × 50 cm, 2 μm C18) using a gradient of 7% to 35% solvent B (90% acetonitrile in 0.1% formic acid) over 110 minutes. MS/MS analysis was carried out in data-dependent mode. In MS scan, precursor ions were analyzed in the Orbitrap analyzer from 350–1600 m/z with 50 ms IT and 120,000 resolution. Top 15 precursor ions were sequentially isolated by quadrupole mass filter using 1.6 m/z isolation width and fragmented using high energy collision-induced dissociation (HCD) method with 32% NCE. Fragment ions were analyzed in the Orbitrap analyzer with 100 ms IT and 45,000 resolution. Dynamic exclusion was enabled during the run with a 40-sec exclusion duration.

### Data analysis

Proteome Discoverer version 2.1.0.81 (Thermo Fisher Scientific, Bremen, Germany) was used for analyzing the raw files. Precursor mass range of 350–2000 Da and signal to noise ratio of 1.5 was the criteria used for the generation of peak lists. Sequest and Mascot search algorithms were used for database searches against the human Refseq 89 protein database containing common contaminants. Search parameters included a maximum of one missed cleavage, 10 ppm precursor mass tolerance, and 0.05 Da fragment ion tolerance. Carbamido-methylation at cysteine, iTRAQ 4-plexmodification at N-terminus of a peptide, and lysine were set as fixed modification while oxidation of methionine as variable modifications. The Decoy database was used to calculate the false discovery rate (FDR) with a cut-off of 1% at the PSM level [41]. Integration tolerance of 30 ppm around reporter ion peaks was used for calculating the reporter ion intensities.

### Statistical analysis

Perseus (version 1.6.2.2) was used for all the statistical analyses. The expression matrix containing abundance values across all the replicates were used for the PCA analysis. ANOVA based statistical analysis was used to perform the clustering in the heatmaps (*p* value =< 0.05). The differentially regulated proteins in each subtype of FELs were identified using ± 1.5-fold cut-off.

### Gene ontology term analysis

The functional characterization of the differentially expressed proteins was carried out based on gene ontology terms using DAVID (The Database for Annotation, Visualization, and Integrated Discovery) Bioinformatics Resources 6.8. (https://david.ncifcrf.gov/)[42].

### Validation of differentially expressed proteins by Immunohistochemistry

Sections were deparaffinized in xylene, washed in gradients of ethanol, and rehydrated. Heat-induced epitope (antigen) retrieval was performed by boiling slides with two different buffers, 10 mM Tris base, 1mM EDTA pH 9.0 for HTRA1, MUCL1, VEGFD, Ki67, C-myc, retinoic acid receptor-α, and 10 mM sodium citrate buffer pH 6.0 for UCHL1, PIP, Vimentin, CD44, Cyclin D for 30 min each at 100°C. Slides were then cooled for 15 min before washing with 1X TBS. Blocking of endogenous protein was done by using 10% BSA in 1X TBS for 2 hrs at room temperature. Slides were then incubated with primary antibodies HTRA1, MUCL1 and VEGFD, UCHL1, PIP, Vimentin, C-myc, Ki67, CD44, cyclin D retinoic acid receptor-α, all at 1:100 dilutions at 4°C overnight in a humid chamber. Next day slides were washed three times with 1X TBST (0.1% Triton- X) for 15 min and blocked with 3% H2O2 in methanol for 30 min to quench the endogenous peroxidase activity of the sections. Slides were washed first with water and then with TBS and were then incubated with anti-rabbit HRP conjugated secondary IgG (Abcam ab97051) for 2 hrs at room temperature in a humid chamber. After incubation, slides were washed three times with 1X TBST for 15 min and diaminobenzidine (DAKO Envision) was used (1 min) as a chromogenic agent for color development. After water wash, slides were stained with Gills Hematoxylin Mod III and was mounted. Negative controls of IHCs were prepared for each set of sections by replacing the primary antibody with TBS.

## SUPPLEMENTARY MATERIALS





## Abbreviations

DTT: Dithiothreitol
FADs: Fibroadenomas
FDR: False discovery rate
FELs: Fibroepithelial lesions, IAA: Iodoacetamide
iTRAQ: isobaric tags for relative and absolute quantification
PTs: Phyllode Tumors
