# Supplementary material for: Quantitative proteome profiling stratifies fibroepithelial lesions of the breast
Source: Oncotarget. 2021 Mar 2;12(5):507–18. doi: 10.18632/oncotarget.27889 (PMC7939526; doi:10.18632/oncotarget.27889)
Supplement: Supplementary file 1 [file oncotarget-12-507-s001.pdf]

Quantitative proteome profiling stratifies fibroepithelial lesions of the breast

SUPPLEMENTARY MATERIALS

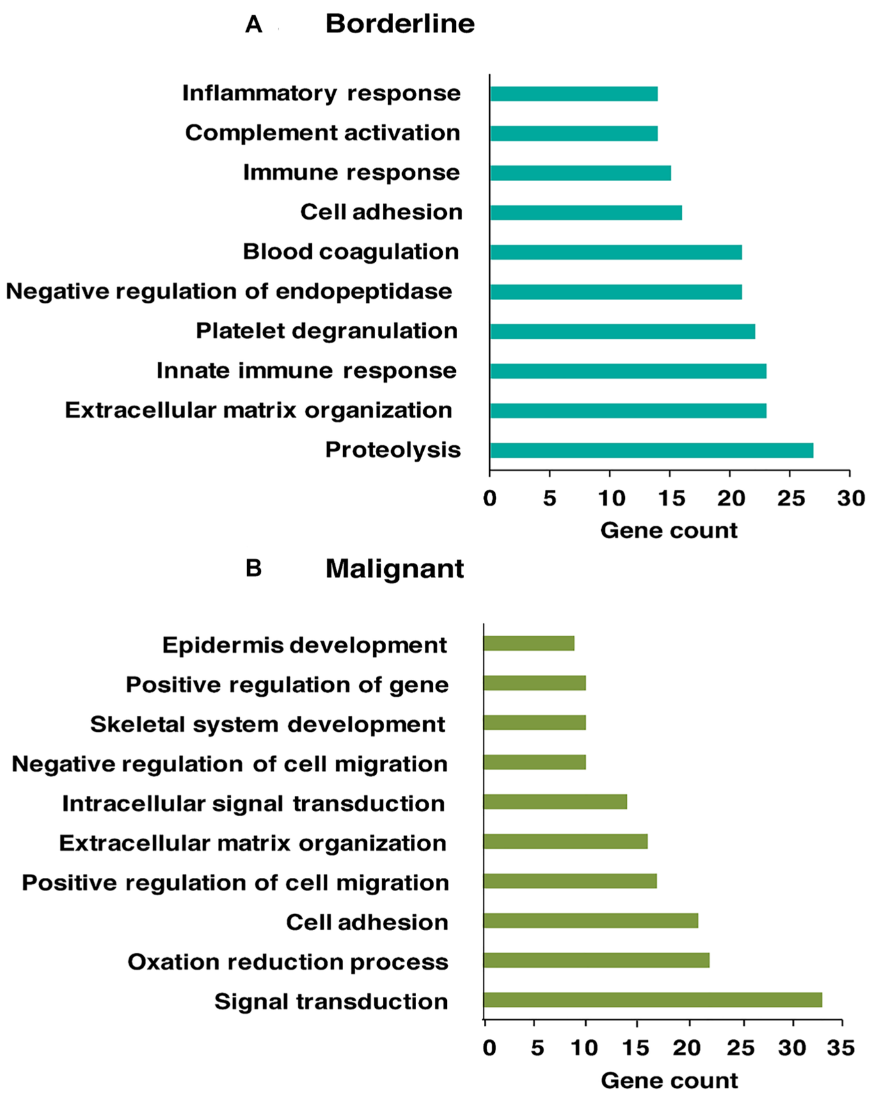

Supplementary Figure 1: Enriched gene ontology terms of deregulated proteins in (**A**) borderline and (**B**) malignant PTs. The y-axis depicts the biological process and x-axis shows the number of genes associated with each process.

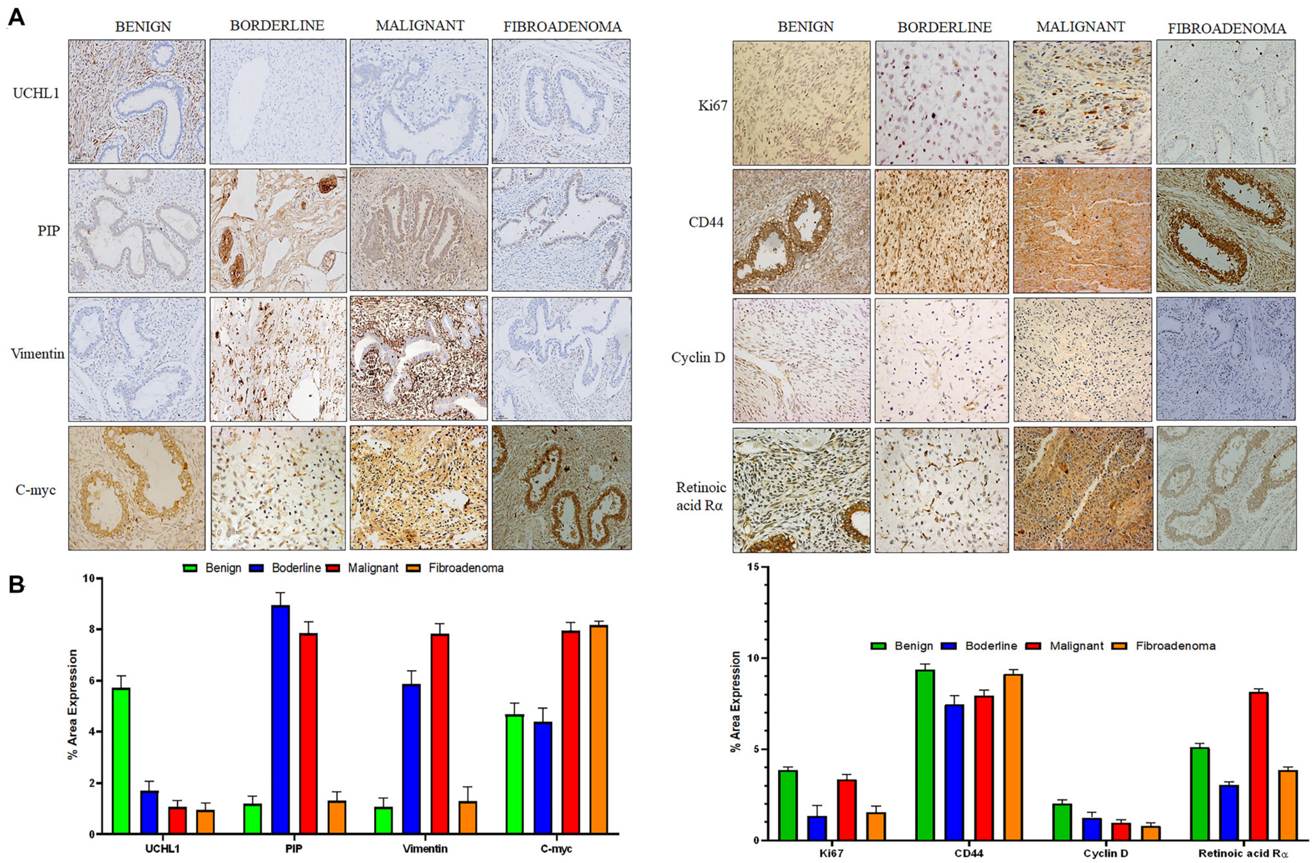

Supplementary Figure 2: (**A**) Immunohistochemical staining of different proteins involved in FELs pathogenesis in biological replicates of benign, borderline, malignant and fibroadenoma, (**B**) Percentage area expression of various proteins in different types of breast epithelial lesions using Image J (IHC toolbox).

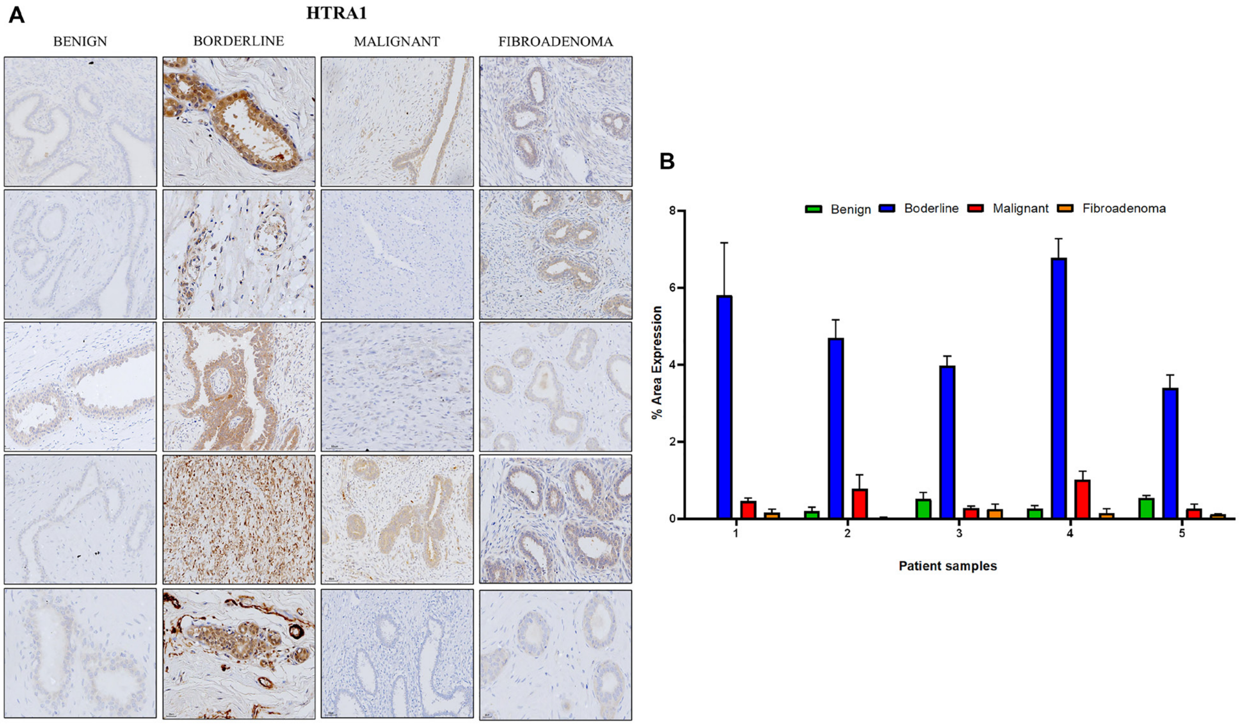

Supplementary Figure 3: (**A**) Immunohistochemical staining of HTRA1 in five independent biological replicates of benign, borderline, malignant and fibroadenoma, (**B**) Percentage area expression of HTRA1 in different types of breast epithelial lesions using Image J (IHC toolbox).

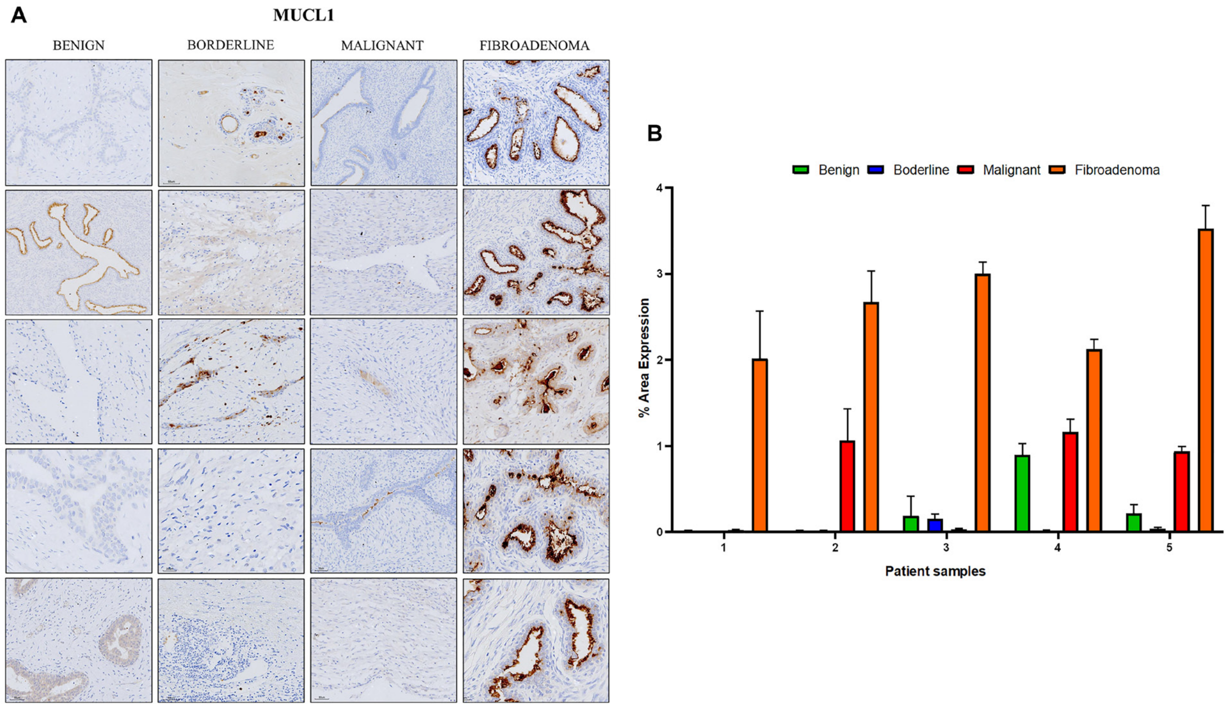

Supplementary Figure 4: (**A**) Immunohistochemical staining of MUCL1 in five independent biological replicates of benign, borderline, malignant and fibroadenoma, (**B**) Percentage area expression of MUCL1 in different types of breast epithelial lesions using Image J (IHC toolbox).

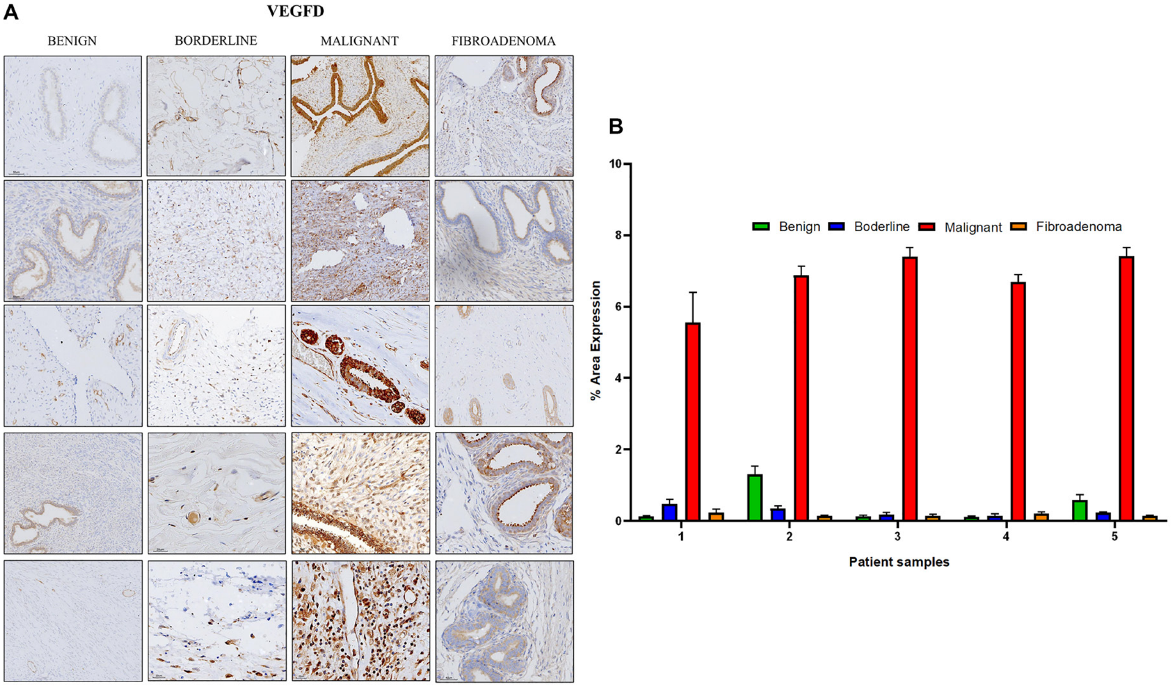

Supplementary Figure 5: (**A**) Immunohistochemical staining of VEGFD in five independent biological replicates of benign, borderline, malignant and fibroadenoma, (**B**) Percentage area expression of VEGFD in different types of breast epithelial lesions using Image J (IHC toolbox).

Supplementary Table 1: Details of the samples used for proteomics study

S.No.	Sample ID	Category	Scrolls used
1	832/16	Benign	2
2	9464-A/16	Benign	2
3	10615-B/16	Benign	2
4	12456-FB/16	Benign	2
5	12591/16	Benign	2
6	152-A1/16	Borderline	2
7	10813/15	Borderline	2
8	14414/17	Borderline	2
9	3283-FB/14	Borderline	2
10	9828-A/15	Borderline	2
11	6811-A5/15	Malignant	2
12	13698-A/15	Malignant	2
13	14445/15	Malignant	2
14	1376-FB	Malignant	2
15	22188-A/15	Malignant	2
16	1285/16	Fibroadenoma	2
17	13602/16	Fibroadenoma	2
18	1244-A/16	Fibroadenoma	2
19	13297/16	Fibroadenoma	2
20	9464-B/15	Fibroadenoma	2

Supplementary Table 2: Master list of total identified proteins and their median abundance values in fibroepithelial lesions. See Supplementary Table 2

Supplementary Table 3: Master list of total proteins and their fold change values in fibroepithelial lesions. See Supplementary Table 3

Supplementary Table 4: List of differentially expressed proteins in FAD vs BE. See Supplementary Table 4

Supplementary Table 5: List of differentially expressed proteins in BT vs BE. See Supplementary Table 5

Supplementary Table 6: List of differentially expressed proteins in MT vs BE. See Supplementary Table 6
